# Therapeutic implications of nanoencapsulated *M. avium* / HIV drugs against experimental tuberculosis in mice

**DOI:** 10.1186/1471-2334-12-S1-P47

**Published:** 2012-05-04

**Authors:** Tapinder Grewal, Sadhna Sharma

**Affiliations:** 1 Department of Biochemistry, Postgraduate Institute of Medical Education and Research, Chandigarh, India

## Background

Therapeutic management of *Mycobacterium avium* infection is inadequate due to patient non-compliance, lengthy treatment regimen, multidrug associated toxic side-effects etc. particularly in AIDS patients who are at a greater risk of developing mycobacterial infection. This study was designed to evaluate chemotherapeutic potential of poly D, L-lactide-co-glycolide nanoparticles against *M. avium* infection in mice.

## Methods

Drug loaded nanoparticles were prepared by double emulsification and characterized for their size, surface morphology and sustained drug release. Pharmacokinetics of free and nanoencapsulated drugs were evaluated after single oral dose administration and therapeutic efficacy was assessed in *M. avium* infected mice after 4 weeks of chemotherapy.

## Results

Sustained release of various drugs was observed for 5-7 days as compared to 24h for free drugs in plasma and various tissues. Eight weeks of chemotherapy resulted in significant clearance of bacilli from lungs and spleen of *M. avium* infected mice as compared to untreated controls. 8 doses of PLGA nanoencapsulated *M. avium* drugs depicted an equivalent therapeutic effect as that of 56 doses of daily administered oral free drugs which was evident from cfu enumeration data and lung histopathology. Furthermore, nanoencapsulation was observed to lessen the adverse drug interactions between anti-retroviral and anti-*M. avium* drugs.

## Conclusion

PLGA nanoparticle based drug delivery system showed great potential to produce sustained release of anti-HIV / *M. avium* drugs. These studies hold promise to reduce the frequency of drug dosages as well as alleviate adverse drug interactions during the course of *M. avium* and HIV therapy.

